# Natural variation of life‐history traits, water use, and drought responses in Arabidopsis

**DOI:** 10.1002/pld3.35

**Published:** 2018-02-01

**Authors:** John N. Ferguson, Matt Humphry, Tracy Lawson, Oliver Brendel, Ulrike Bechtold

**Affiliations:** ^1^ School of Biological Sciences University of Essex Colchester UK; ^2^ Advanced Technologies Cambridge Cambridge Science Park Cambridge UK; ^3^ EEF, INRA, Université de Lorraine Champenoux France; ^4^ Present address: Institute for Genomic Biology University of Illinois at Urbana‐Champaign Urbana IL USA; ^5^ Present address: British American Tobacco Cambridge Science Park Cambridge UK

**Keywords:** *Arabidopsis thaliana*, biomass, drought sensitivity, leaf‐level water‐use efficiency, photosynthesis, vegetative water use, water use

## Abstract

The ability of plants to acquire and use water is critical in determining life‐history traits such as growth, flowering, and allocation of biomass into reproduction. In this context, a combination of functionally linked traits is essential for plants to respond to environmental changes in a coordinated fashion to maximize resource use efficiency. We analyzed different water‐use traits in Arabidopsis ecotypes to identify functionally linked traits that determine water use and plant growth performance. Water‐use traits measured were (i) leaf‐level water‐use efficiency (WUE_*i*_) to evaluate the amount of CO_2_ fixed relative to water loss per leaf area and (ii) short‐term plant water use at the vegetative stage (VWU) as a measure of whole‐plant transpiration. Previously observed phenotypic variance in VWU, WUE_*i*_ and life‐history parameters, highlighted C24 as a valuable ecotype that combined drought tolerance, preferential reproductive biomass allocation, high WUE_*i*_, and reduced water use. We therefore screened 35 Arabidopsis ecotypes for these parameters, in order to assess whether the phenotypic combinations observed in C24 existed more widely within Arabidopsis ecotypes. All parameters were measured on a short dehydration cycle. A segmented regression analysis was carried out to evaluate the plasticity of the drought response and identified the breakpoint as a reliable measure of drought sensitivity. VWU was largely dependent on rosette area, but importantly the drought sensitivity and plasticity measures were independent of the transpiring leaf surface. A breakpoint at high rSWC indicated a more drought‐sensitive plant that closed stomata early during the dehydration cycle and consequently showed stronger plasticity in leaf‐level WUE_*i*_ parameters. None of the sensitivity, plasticity, or water‐use measurements were able to predict the overall growth performance; however, there was a general trade‐off between vegetative and reproductive biomass. PCA and hierarchical clustering revealed that C24 was unique among the 35 ecotypes in uniting all the beneficial water use and stress tolerance traits, while also maintaining above average plant growth. We propose that a short dehydration cycle, measuring drought sensitivity and VWU is a fast and reliable screen for plant water use and drought response strategies.

## 1 INTRODUCTION

Plant growth, survival, and reproduction are life‐history traits that are known to respond to environmental fluctuations leading to variations in the amount of resources available to a plant (Anderson, 2016). Life‐history strategies rely on trade‐offs between survival, growth, and/or reproductive performance, largely driven by the ability of plants to acquire net carbon and to allocate these resources into biomass and fitness (Salguero‐Gómez et al., 2016). Water is clearly a vital resource to all aspects of plant physiology and growth (Boyer, 1982; Lambers, Chapin, & Pons, 1998), and water limitation is often a major constraint to plant survival and productivity, by restricting the acquisition of carbon (Claeys & Inze, 2013; Schulze & Hall, 1982; Sinclair & Rufty, 2012). Plants adapt to drier environments by reducing the transpiring leaf surface (i.e., smaller leaves) or through changes in relative rates of gas exchange, maximizing the ratio of carbon gain to water loss, defined as water‐use efficiency (WUE). The availability of water is therefore critical in shaping growth and reproductive allocation of plants, and if water availability is poor, survival trade‐off costs will result in reduced reproductive fitness (Claeys & Inze, 2013; von Euler, Ågren, & Ehrlén, 2012; Sletvold & Ågren, 2015). Climate change is predicted to increase the frequency and severity of future incidents of drought (Famiglietti & Rodell, 2013), and the ecological impact will depend on the extent to which plants can respond to these changing conditions. Recurrent periods of drought impose strong selective pressures on populations to evolve different life‐history strategies for adaptation to such conditions, including the emergence of adaptive genes through dispersal from within the species range, and selection on pre‐existing genetic variation (Aitken, Yeaman, Holliday, Wang, & Curtis‐McLane, 2008).

In the context of plant water use, different levels of WUE are commonly used to assess aspects of plant growth performance in relation to water use. Whole‐plant water‐use efficiency also known as transpiration efficiency (TE, Tanner & Sinclair, 1983) is defined as the total biomass produced per unit of water transpired. At the leaf level, WUE is defined as the net amount of CO_2_ fixed per given unit of water transpired (*A*/*E*), referred to as instantaneous water‐use efficiency (WUE_*i*_, Field, Merino, & Mooney, 1983). WUE_*i*_ is considered to be an important factor in plant water use, as it relates equally to water loss by transpiration and net carbon gain achieved via gas exchange, potentially impacting on the production of biomass (Long, Marshall‐Colon, & Zhu, 2015; Steduto, Hsiao, & Fereres, 2007). In recent years, high leaf‐level WUE has been considered an important trait to minimize the loss of water in many different plants species (Blum, 2009; Sinclair & Rufty, 2012; Vadez, Kholova, Medina, Kakkera, & Anderberg, 2014). This is because the relationship between leaf and plant‐level WUE parameters is based on the principle that biomass accumulation is driven by carbon assimilation, modulated by nighttime respiration, while water use is mainly driven by stomatal transpiration.

WUE is often referred to as a drought adaptation trait (Comstock et al., 2005; Condon, Richards, Rebetzke, & Farquhar, 2004; McKay et al., 2008), but actually only evaluates how much water a plant needs to produce biomass. This is due to the shape of the *A/g*_s_ correlation, where water‐use efficiency can increase during drought stress when stomata close, especially when *A* is not yet proportionally affected (Easlon et al., 2014; Gilbert, Holbrook, Zwieniecki, Sadok, & Sinclair, 2011; Meinzer, Goldstein, & Jaimes, 1984). This is the case in Arabidopsis where within‐species variation in water‐use efficiency is predominantly driven by variation in stomatal conductance with relatively little evidence of variation in photosynthetic capacity (Easlon et al., 2014). This suggests that overall plant water use will be the main driver of TE*,* and as a consequence, improvements in leaf‐level WUE may be realized at the expense of reproductive growth (Condon, Richards, Rebetzke, & Farquhar, 2002; Morison, Baker, Mullineaux, & Davies, 2008).

At the plant level, water use can be monitored by determining gravimetric relative soil water content over a defined period time (Bechtold et al., 2010, 2013; Easlon et al., 2014), or through the use of automated weighing and watering platforms (Halperin, Gebremedhin, Wallach, & Moshelion, 2017; Ryan et al., 2016; Tisne et al., 2013). By monitoring the decline in gravimetric soil water content in pot‐based experiments, we are able to calculate the average rosette water use as the slope of the linear regression (Bechtold et al., 2010). We have subsequently named this parameter vegetative water use (VWU), which represents the “un‐normalized” absolute rosette water use.

There has been a longstanding argument that the “effective use of water” is a much more important parameter to consider especially when looking at plant productivity. This concept suggests that maximal soil moisture capture for transpiration and decreased water use are important for maximizing plant productivity under limited water supplies (Blum, 2009; Polania, Poschenrieder, Beebe, & Rao, 2016). The parameter VWU quantifies water use at the vegetative growth stage, and while VWU may not represent “effective use of water” or life‐time water use, it allows us to establish direct relationships between water transpired from the soil, the plants’ physiology, and its growth performance.

The phenotypic variance in VWU, life‐time water use and life‐history parameters previously observed in few ecotypes (Bechtold et al., 2010), suggests that some are better at converting available water into biomass compared to others. However, monitoring life‐time plant water use in combination with biomass production is a relatively low‐throughput trait, which has inevitably meant that this parameter has received limited attention in Arabidopsis (Bechtold et al., 2010, 2013). From these limited studies, we observed that the ecotype C24 manages to combine drought tolerance, preferential biomass allocation into reproductive growth, high WUE_*i*_ under well‐watered conditions, as well as low VWU (Bechtold et al., 2010), and the question arose whether this combination of traits existed more widely across Arabidopsis ecotypes, highlighting the need for a larger scale screen.

Therefore, the primary aim of this study was to investigate the relationship between VWU, drought tolerance, and plant growth performance in comparison with the more commonly used leaf‐level WUE measurements WUE_*i*_ (*A*/*E*) in 35 Arabidopsis ecotypes. The objectives for the ecotype screen were as follows: (i) to develop the short dehydration cycle as a fast and reliable screen for plant water use and drought response strategies; (ii) to validate the screen by analyzing the natural variation for VWU, leaf‐level WUE*,* and plant growth performance in comparison to the ecotype C24; and (iii) to assess the drought sensitivity and drought response strategy in the selected Arabidopsis ecotypes.

## 2 MATERIALS AND METHODS

### 2.1 Plant material

**Figure 1:**
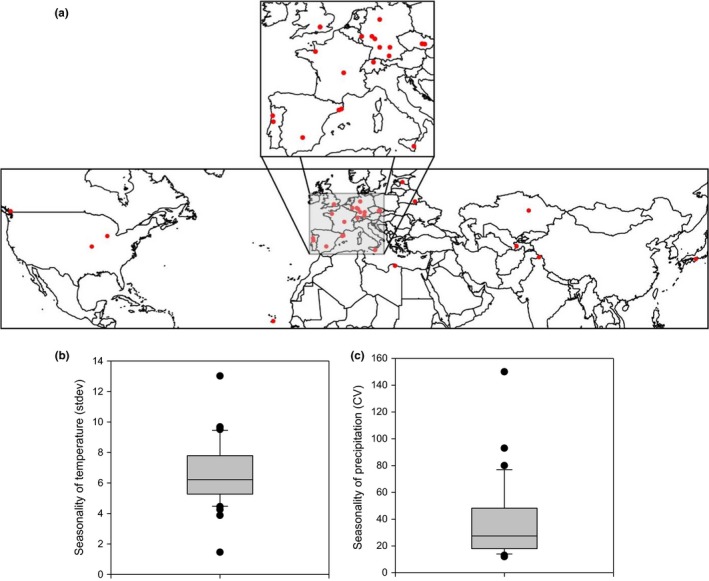
Geographic distribution and climatic history variation of the 35 Arabidopsis ecotypes comprising the present study. (a) Geographic site of origin of all ecotypes comprising this study, as indicated by the red circles. (b) The variation in temperature seasonality at the site of origin of the 35 ecotypes. (c) The variation in annual mean precipitation at the site of origin of the 35 ecotypes. Seasonality of temperature (stdev × 100) and precipitation is according to BIOCLIM database. Outliers are data points outside the 1.5 ×  interquartile range both above the upper quartile and below the lower quartile

Seed for all ecotypes comprising this study was obtained from the Nottingham Arabidopsis Stock Centre (NASC; Scholl, May, & Ware, 2000). This study included 35 Arabidopsis ecotypes that represent a wide distribution across the Northern Hemisphere (Figure 1, Table S1).

### 2.2 Growth conditions

Plants were grown in peat‐based compost (Levington F2 + S, The Scotts Company, Ipswich, UK), 6‐cm‐diameter pots (0.12 L) in two different environments. Seeds were sown in soil and stratified for 4 days in the dark at 4°C, before being transferred to the controlled growth rooms. In the controlled growth room, plants were kept in an 8/16‐hr light/dark cycle at a photosynthetically active photon flux density (PPFD) of 120 μmol m^−2^ s^−1^, at a constant relative humidity of 60% (VPD at 1 kPa), and 23°C.

Within the glasshouse, the environmental conditions were variable, as temperature and external light cycles fluctuated during the experimental periods. In the glasshouse, supplemental lighting was maintained at a minimum PPFD threshold of ~150 μmol m^−2^ s^−1^ at plant level for a 12‐hr day. Plants were kept well‐watered, except during the short dehydration experimental period, and their positions were changed every 3 days.

### 2.3 Short dehydration experiment and trait parameter assessed

Plants were grown in the growth chamber for the determination of VWU. Gravimetric relative soil water content (rSWC) was calculated based on the volume of H_2_O required (on average ~ 95 ml) to saturate the identical amount of soil in each pot. This allowed us to convert between rSWC (%) and ml H_2_O per pot in order to calculate VWU (ml/day). At 54 days, all plants were well‐watered and were left to progressively dry to ~20% rSWC (Bechtold et al., 2010), at which point they were rewatered and transferred from the controlled environment room to the glasshouse for flowering time determination and seed production. VWU was calculated as the slope of the linear regression of the drying rate across the entire drying period (Figure S1a). The linear model was evaluated for goodness of fit based on residual analysis and diagnostic plots produced as part of the lm() function in R. VWU therefore evaluates the short‐term water use during the period of dehydration, and due to the linear regression, it can be viewed as water use per day. Within the glasshouse, plants were kept well‐watered for the remaining experimental duration. At the point of opening of the final flower, plants were bagged and allowed to dry out for harvesting. During harvest, the vegetative (rosette) and reproductive (stalks, pods, and seeds) biomass components were separated (Bechtold et al., 2010, 2013). Prior to the onset of the short dehydration period, RGB photographs of all plants were taken and the total rosette area of individual plants was determined using the ImageJ (http://www.imagej.nih.gov/ij/) analysis software. To determine that a short dehydration period does not lead to early flowering, a selection of ecotypes with different drying rates was either subjected to the short dehydration period or maintained well‐watered (Figure S1b,c). Flowering time and biomass parameters were collected as described above.

Instantaneous (snapshot) photosynthetic measurements were taken through infrared gas exchange analysis using portable infrared gas exchange systems (CIRAS‐2; PP Systems, Amesbury, MA, USA). All snapshot photosynthesis measurements were taken on fully expanded upper rosette leaves and taken at three points during progressive dehydration (90%, 40%, and 20% rSWC). All readings were taken at current atmospheric [CO_2_] and under PPFD of 150 μmol m^−2^ s^−1^. Readings were recorded when rates of photosynthetic carbon assimilation (*A*), and stomatal conductance (*g*_s_), were steady (ca. 2–3 min). Instantaneous water‐use efficiency (WUE_*i*_) was calculated using carbon assimilation (*A*) and rate of transpiration (*E*) as *A/E*.

### 2.4 Segmented regression analysis and calculations of plasticity

We used the Davies test (Davies, 2002) and segmented regression analysis as part of the segmented package in R (Muggeo, 2017) in order to test (i) for a significant difference in slope parameter and (ii) for the breakpoint in the regression. This analysis produced the breakpoint in the drying period and the slopes before (stage 1) and after (stage 2) the breakpoint (Figure S2). VWU plasticity was calculated as the slope before the breakpoint (stage 1)—slope after breakpoint (stage 2)/slope before breakpoint (stage 1). Similarly, the plasticities of *g*_s_ and WUE_*i*_ were calculated based on stage 1 (90% rSWC—40% rSWC/90% rSWC) and stage 2 (40% rSWC—20% rSWC/20% rSWC) snapshot measurements.

### 2.5 Data analyses

Unless stated, all statistical analyses were performed within the R software environment for statistical computing and graphics (R Core Team, 2015). The short dehydration experiments were temporally divided into seven experimental blocks (Table S1). Every experimental block contained the Col‐0 and C24 ecotypes. A randomized block design was used for each experimental block, where plants were grown at random positions within the controlled growth room to reduce variability within each experiment. Shapiro–Wilks tests were performed for all parameters to test for normal distribution. For all parameters and depending on trait distribution, either parametric one‐way analysis of variance (ANOVA) or nonparametric Kruskal–Wallis test was performed for comparison of means testing of all ecotypes across experimental blocks, as well as for just the Col‐0 and C24 ecotypes across experimental blocks.

To account for detected experimental block effects, best linear unbiased predictors (BLUPs) of ecotype means were calculated according to Merk et al. (2012). BLUPs provide robust predictions of the genotype effect, while accounting for random effects (Lynch & Walsh, 1998). Predicted means were subsequently calculated by adding the BLUPs to population means for each trait. Predicted means were utilized for all subsequent comparison and correlation/regression analyses.

For all traits, the among genotypic (ecotype) variation (*V*_G_) and the phenotypic variation (*V*_P_) were determined with general linear mixed models (GLMM). This was achieved via the lmer() function from the lme4 R package (Bates, Maechler, & Bolker, 2013). Ecotype and experimental block were treated as random effects for all GLMMs. Broad‐sense heritability (*H* ^2^) was calculated as *V*_G_/*V*_P_, and significance was established by computing analysis of deviance tables for a GLMM where ecotype was included as a random‐effect predictor and one that did not include ecotype as a predictor.

Phenotypic correlations were calculated as the standard Pearson product–moment correlation among predicted means (Easlon et al., 2014; Lau, Shaw, Reich, Shaw, & Tiffin, 2007; McKay, Richards, & Mitchell‐Olds, 2003; McKay et al., 2008). We employed a sequential Bonferroni correction to all *p*‐values in the correlation matrix to insure against the risk of false positives (Holm, 1979).

A Euclidean distance matrix between all pairs of genotypes was computed based on three traits (*WUE*_*i*_ 20%, VWU, and HI), and the phenogram of Arabidopsis ecotypes was constructed based on the unweighted pair group method with arithmetic mean (UPGMA) using the dist() and hclust() functions in R.

Principal component analysis (PCA) was conducted using the prcomp() function in R on the life‐history trait datasets of 35 ecotypes. The Kaiser–Guttman rule was used to determine significant principle components (Kaiser, 1991).

## 3 RESULTS

Phenotypic variation of VWU was investigated in 35 randomly selected Arabidopsis accessions that originated from a range of different habitats across the Northern Hemisphere (Figure 1a,b; Table S1). VWU was assessed by performing a short dehydration experiment followed by segmented regression analysis (see Section 2). This allowed the determination of VWU as the slope of the linear regression over the entire drying period (Figure S1a), as well as the slope parameters and breakpoint between early‐ and late‐stage drying (Figure S2a). Physiological snapshot measurements (*A*,* g*_s_, *E,* WUE_*i*_ *(A/E))* were taken at three points (90%, 40%, and 20% rSWC) during the dehydration episode. Plants were otherwise maintained well‐watered throughout the entire growth period, and we subsequently determined biomass parameters (vegetative and reproductive biomass) and flowering time. Importantly, the short dehydration cycle applied to determine VWU did not initiate an active drought escape mechanism by inducing early flowering, nor did it detrimentally impact on seed biomass compared to well‐watered control plants, as assessed on a subset of 12 ecotypes (see Section 2; Figure S1b,c).

### 3.1 Phenotypic variation in photosynthesis, daily water use, flowering time, and biomass

The 35 accessions used in the short dehydration experiment were grown in seven separate experimental blocks. Each experimental block contained an average of five ecotypes, with Col‐0 and C24 grown in all experimental blocks to assess environmental variation across blocks (Table S1). We identified significant experimental block effects for all traits (Table S2). This variation between experiments is in line with previous results where within and across laboratories variation was demonstrated, even if plants were grown in identical pots, soil, and environmental conditions (Massonnet et al., 2010). Consequently, BLUPs were extracted and predicted means for each genotype were calculated to control for random experimental block effects. Pearson product–moment correlations were calculated between arithmetic means and predicted means, which demonstrated a significant positive association (Table S3).

**Figure 2:**
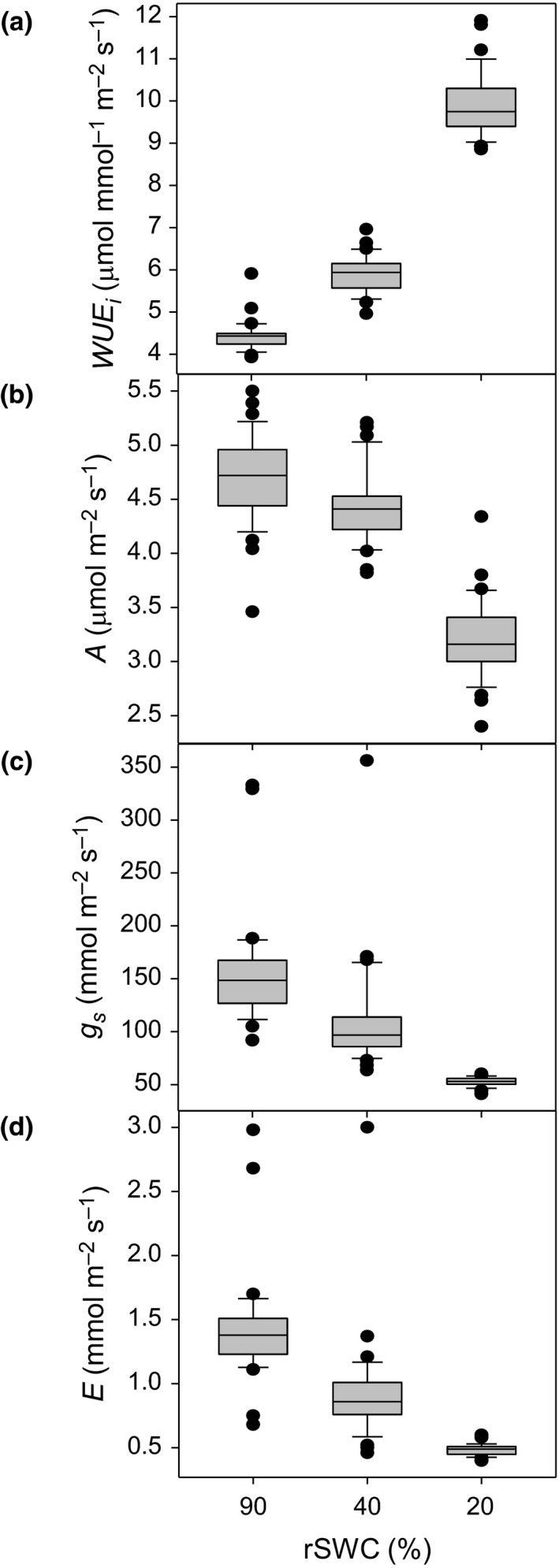
Variation in photosynthesis during the short dehydration (*SD*) period. Boxplots demonstrating the variation (a) instantaneous water‐use efficiency (WUE_*i*_; *A*/*E)*, (b) carbon assimilation (*A*)*,* (c) stomatal conductance (*g*_s_)*,* and (d) transpiration *(E)* at 90%, 40%, and 20% rSWC. For all boxplots, the bottom and top boxes denote the 25th and 75th percentiles, respectively. The central band is the 50th percentile. Whiskers extend to the most extreme data points which are no more than 1.5 ×  the length of the upper or lower segment away from the respective segment. Outliers are data points outside the 1.5 ×  interquartile range both above the upper quartile and below the lower quartile

Positive phenotypic correlations between stomatal conductance (*g*_s_) and carbon assimilation (*A*) were observed under well‐watered (90% rSWC) and water‐limited conditions (40% and 20% rSWC; Table S4), while negative correlations occurred between traits in evolutionary constraint, such as WUE_*i*_, transpiration (*E*) and *g*_s_ (Table S4). The leaf‐level WUE_*i*_ at 90%, 40%, and 20% rSWC was not phenotypically linked to life‐history traits such as flowering time and aboveground biomass (Figure S3; Table S4). There was substantial natural variation for *A*,* g*_s*,*_ and *E* under well‐watered conditions (90% rSWC; Figure 2). The variation in *E* and *g*_s_ reduced with increasing dehydration (Figure 2c,d), but *A* maintained the same level of variation at 20% rSWC (Figure 2b, Table 1). The increase in WUE_*i*_ at 40% and 20% rSWC (Figure 2a) was primarily driven by a reduction in *E* and *g*_s_ (Table 1), yet the increase in variation in *WUE*_*i*_ at 20% rSWC appeared to be due to variation in *A* (Figure 2b).

**Table 1:** Broad‐sense heritability (*H* ^2^) of 35 phenotypic traits. Significant heritabilities (Sig.) are indicated as *p < .025, **p < .01, ***p < .001. *V*_G_, genetic variation; *V*_P_, phenotypic variation

Trait	Population mean (*SE*)	*V*_G_	*V*_P_	*H* ^2^	Sig.
Rosette area	27.40 (0.71)	73.36	136.57	0.54	***
VWU	9.19 (0.06)	1.22	2.99	0.41	***
90% *A*	4.50 (0.08)	0.25	2.72	0.09	***
90% *E*	1.35 (0.06)	0.17	1.15	0.15	***
90% WUE_*i*_	4.65 (0.17)	0.83	10.67	0.08	**
90% *g*_s_	143.66 (4.80)	2,660.00	8,581.00	0.31	***
90% *C*_*i*_	297.76 (2.39)	99.74	1,959.47	0.05	***
40% *A*	4.35 (0.09)	0.23	3.22	0.07	***
40% *E*	0.87 (0.29)	0.15	1.12	0.14	***
40% WUE_*i*_	6.18 (0.18)	0.57	11.18	0.05	*
40% *g*_s_	102.59 (3.49)	2,120.10	4,397.70	0.48	***
40% *C*_*i*_	291.84 (2.51)	137.00	2,190.80	0.06	***
20% *A*	3.10 (0.07)	0.24	2.31	0.10	***
20% *E*	0.45 (0.02)	0.00	0.10	0.04	**
20% WUE_*i*_	10.14 (0.53)	0.53	11.36	0.05	n.s.
20% *g*_s_	49.43 (1.89)	54.37	1,450.53	0.04	**
20% *C*_*i*_	266.59 (5.02)	1,381.70	13,979.00	0.10	***
Flowering time	72.10 (0.61)	199.48	355.39	0.56	***
Rosette leaves at flowering	56.55 (0.78)	396.90	705.40	0.56	***
Rosette biomass	0.30 (0.04)	0.00	1.51	0.00	n.s.
Chaff biomass	0.40 (0.01)	0.04	0.10	0.39	***
Seed yield	0.10 (0.00)	0.00	0.01	0.19	***
Aboveground biomass	0.87 (0.00)	0.00	0.00	0.24	***
Harvest index (HI)	0.09 (0.00)	0.00	0.01	0.30	***
Breakpoint (%rSWC)	40.2 (0.5)	14.1	239.8	0.05	***
Slope 1	10.89 (0.12)	1.50	16.08	0.093	***
Slope 2	5.70 (0.06)	1.01	3.33	0.30	***
VWU plasticity	0.42 (0.01)	0.005	0.04	0.12	***
Gs plasticity	0.643 (0.01)	0.006	0.07	0.08	***
Gs plasticity stage 1	0.454 (0.01)	0.004	0.056	0.07	***
Gs plasticity stage 2	0.55 (0.01)	0.005	0.058	0.09	***
WUEi plasticity	0.87 (0.01)	0.0004	0.03	0.01	***
WUEi plasticity stage 1	0.45 (0.02)	0.006	0.25	0.02	***
WUEi plasticity stage 2	0	0	0.49	0	ns

We subsequently assessed biomass production and biomass allocation into reproductive structures (harvest index; HI) based on photosynthetic snapshot measurements (Figure S4). We multiplied the per leaf area snapshot photosynthesis measurements at 90% rSWC with the rosette area resulting in an estimate of whole rosette photosynthesis, hereafter termed *A*_(rosette)_ and *g*_s(rosette)_ (Righetti et al., 2007). Neither *A*_(rosette)_ nor *g*_s(rosette)_ significantly correlated with total biomass production or HI (Figure S4a,b). The trait medians were used to subdivide the data into four groups of (i) low *A*_(rosette)_or *g*_s(rosette)_/high HI, (ii) high *A*_(rosette)_or *g*_s(rosette)_/high HI, (iii) low *A*_(rosette)_or *g*_s(rosette)_/low HI, and (iv) high *A*_(rosette)_ or *g*_s(rosette)_/low HI (Figure S4a,b), but no distinct ecotype clusters emerged based on this subdivision. For example, an above average value for *A*_(rosette)_ could lead to either low or high biomass/HI (i.e., Se‐0 and Sq‐1; Figure S4a,b). It was therefore not possible to predict the overall growth performance and biomass allocation based on snapshot measurements of photosynthetic physiology.

### 3.2 Vegetative water use depends on rosette area, but the plasticity of VWU does not

**Figure 3:**
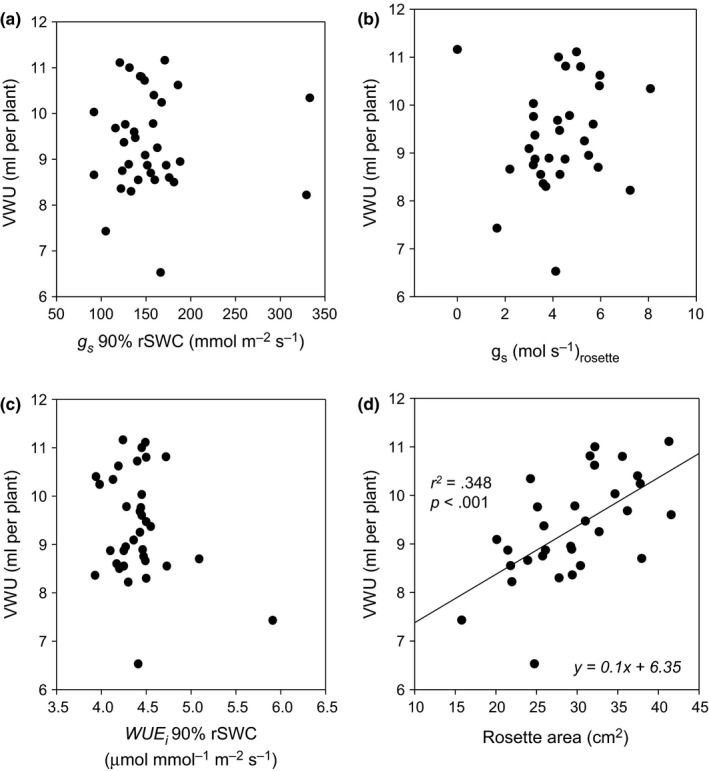
Influence of per leaf area WUE_*i*_ and transpiring leaf surface on VWU (a) Association between vegetative water use (VWU) and stomatal conductance (*g*_s_) at 90% rSWC. (b) Association between VWU and whole rosette stomatal transpiration *g*_s(rosette)._ (c) Association between VWU and WUE_*i*_ *at 90% rSWC*. (d) Association between VWU and rosette area. The line represents the associated linear model. The equation of the linear models is provided along with the associated *r* ^2^ values and *p*‐values

Photosynthetic physiology and stomatal physiology are traits that control the uptake of carbon and loss of water in plants. We therefore wanted to establish whether leaf‐level WUE_*i*_ and stomatal conductance at 90% rSWC are reflective of whole‐plant VWU. VWU was neither correlated with the per leaf area *g*_*s*_ at 90% rSWC (Figure 3a), *g*_s(rosette)_ (Figure 3b) nor WUE_*i*_ at 90% rSWC (Figure 3c). A significant positive correlation of VWU with rosette area highlighted the importance of the leaf surface in water use (Figure 3d, Table S4). This suggested that plant water use relies less on the immediate physiological status of the plant, but more on the transpiring leaf surface.

**Figure 4:**
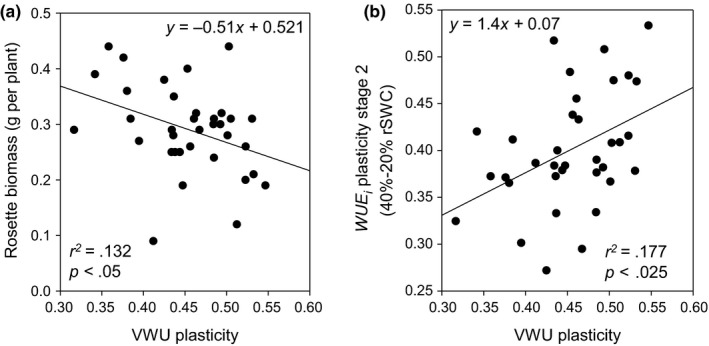
VWU plasticity as a measure of drought response (a) Association between VWU plasticity and rosette biomass. (b) Association between VWU plasticity and WUE_*i*_ plasticity stage 2 (40%–20% rSWC). The line represents the associated linear model. The equations of the linear models are provided along with the associated *r* ^2^ values and *p*‐values

We initially extracted the *R*‐squared (*R* ^2^) values for the linear regression of the full dehydration period (Figure S1a), and the full model resulted in average *R* ^2^ values of .977 (±.0007). Analysis of the residuals and the high *R* ^2^ values suggested that the linear regression was a suitable approximation for VWU. However, based on the visual inspection, the drying curves indicated a bilinear response (Figures S1a and S2), and we therefore used the Davies test (Davies, 2002) to identify differences in slopes across the dehydration period. The Davies test identified two significantly different slopes within the dehydration period across all ecotypes. The subsequent segmented regression model resulted in a small but significant increase in *R* ^2^ to .997 (±.0003), and the automatic detection of breakpoints using segmented regression (Muggeo, 2017) identified the breakpoint and estimated both slopes (before and after the breakpoint). We calculated the VWU, stomatal, and WUE_*i*_ plasticities for stage 1 (90% to 40% rSWC) and stage 2 (40% to 20% rSWC, see Section 2). High values in VWU plasticity indicated greater flexibility in response to water withdrawal, and this negatively correlated with rosette biomass but not rosette area (Figure 4a; Table S4). Importantly, VWU plasticity also positively correlated with WUE_*i*_ plasticity at stage 2 (40%–20% rSWC, Figure 4b; Table S4). This suggests that both WUE_*i*_ plasticity and rosette biomass contributed to the drought response parameter, which was independent of the transpiring leaf surface.

### 3.3 The breakpoint in the dehydration is a measure of drought sensitivity, independent of the transpiring leaf surface

**Figure 5:**
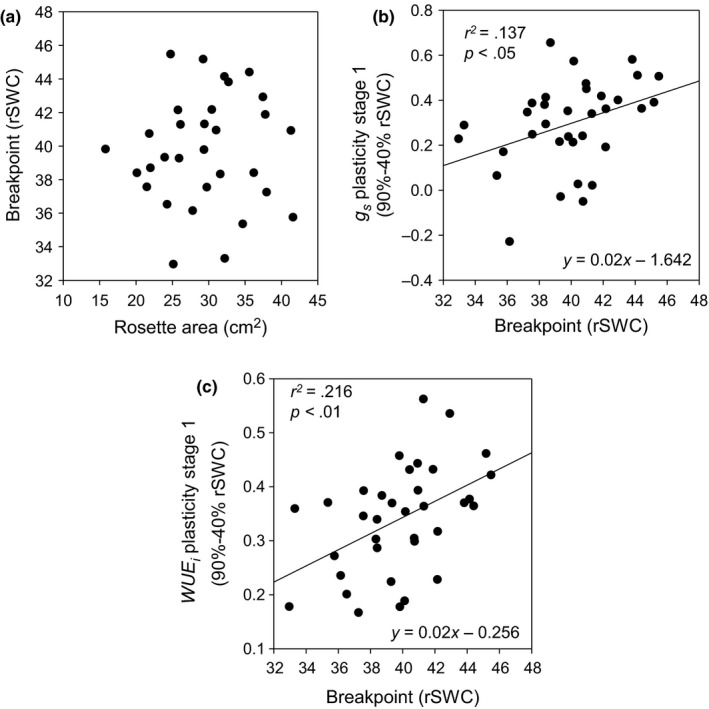
Stomatal responsiveness and drought response plasticity. (a) Association between the rosette area and the breakpoint of the segmented regression analysis. There was no significant relationship between both parameters. (b) Association between the breakpoint of the segmented regression analysis and *g*_s_ plasticity during stage 1 of the dehydration (from 90% to 40% rSWC). (c) Association between the breakpoint of the segmented regression analysis and WUE_*i*_ plasticity during stage 1 of the dehydration (from 90% to 40% rSWC). The lines represent the associated linear model. The equations of the linear model are provided along with the associated *r* ^2^ values and *p*‐values

The average population breakpoint was 40% rSWC during the dehydration (Table 1) and coincided with a recent study in Col‐0 that suggested 40% rSWC as a critical point, at which plants experience substantial transcriptional induction of stress responsive genes (Bechtold et al., 2016; Figure S2b). The breakpoint can therefore be viewed as the threshold below which the plants enter drought stress (Figures S1 and S2a). The breakpoint varied greatly between ecotypes, but importantly it was also independent of the transpiring leaf surface (Figure 5a, Table S4). A significant positive correlation between the breakpoint, stomatal and WUE_*i*_ plasticities during stage 1 (90% to 40% rSWC) indicated that ecotypes entering drought stress at higher rSWC also had greater stomatal and *WUE*_*i*_ responses (Figure 5b,c; Table S4).

### 3.4 Linking water‐use traits with biomass production

It has been suggested that higher seed yield per unit water and therefore increasing HI can be achieved through a reduction of vegetative growth in favor of reproductive growth (French & Schultz, 1984; Blum, 2009). The above‐introduced drought sensitivity and VWU plasticity parameters only evaluated the drought response strategies of ecotypes, but did not allow us to connect plant water use with biomass production. Therefore, we also used the “un‐normalized” absolute value of VWU, as it reflects the water requirements of the plant to produce biomass.

**Figure 6:**
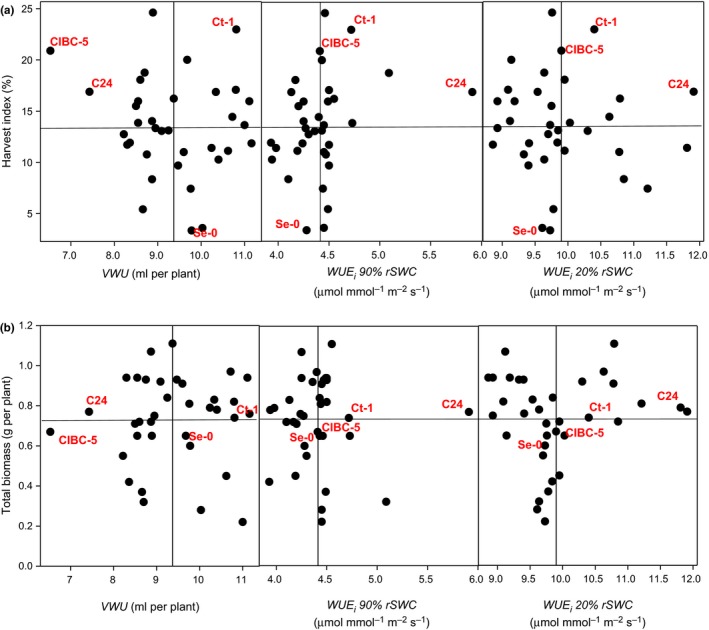
Relationship between water‐use parameters and harvest index. (a) Association between VWU, WUE_*i*_ at 90% and 20% rSWC, and harvest index. (b) Association between VWU, WUE_*i*_ at 90% and 20% rSWC, and total aboveground biomass. Low VWU and high harvest index/total biomass ecotypes are in the upper left quadrant, high WUE_*i,*_ and high harvest index or total biomass ecotypes are in the upper right‐hand quadrants. No significant associations were identified. The lines dividing the space into quadrants represent the median for each trait

**Figure 7:**
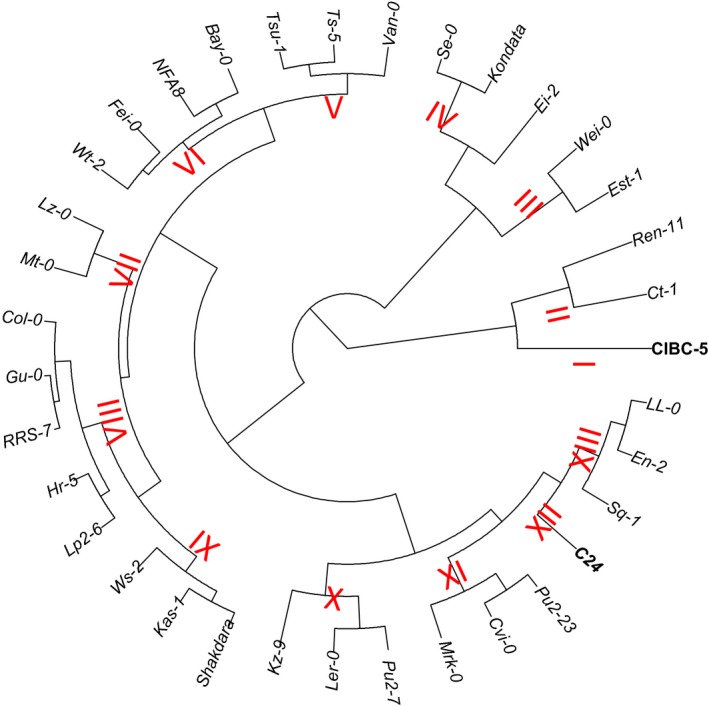
Hierarchical clustering. Clustering of 35 ecotypes by UPGMA based on the Euclidean distance of five traits (WUE_*i*_ 20%, WUE_*i*_ 90%, VWU, total biomass, and harvest index). The thirteen main clusters of ecotypes are indicated in red; summary and rank of the group means are in Table S6. The two single ecotype clusters are highlighted in bold

We assessed whether WUE_*i*_ at 90% and 20% rSWC and/or VWU were useful proxies in predicting HI or total aboveground biomass. There was no significant correlation between VWU, WUE_*i*_ (90% and 20% rSWC), HI, and total biomass (Figure 6a,b). However, C24 stood out as the most water‐use efficient ecotype (high WUE_*i*_ at 20% and 90% rSWC) that also exhibited one of the lowest VWU and above average HI and total biomass (Figure 6a,b; Table 2). In addition, the ranking of ecotypes for individual traits varied greatly and no obvious pattern was visible (Table S5), and consequently, no distinct groups with similar water use and plant growth performance emerged (Figure 6). We therefore performed hierarchical clustering for phenotypic classification of ecotypes based on a combination of five traits related to water use and growth performance (WUE_*i*_ at 90% and 20% rSWC, VWU, total biomass, and HI). The phenogram generated by UPGMA produced thirteen groups, which shared some common characteristics (Figure 7; Table S6). C24 (cluster XII) and CIBC‐5 (cluster I) emerged as single‐member clusters (Figure 7); however, only C24 was able to combine high water‐use efficiency (WUE_*i*_ 20 and 90% rSWC), low VWU and maintain above average biomass production, leading the overall ranking for the combined traits (Tables S5 and S6).

**Table 2:** Predicted means of *WUEi* (μmol (CO_2_) mmol^−1^ (H_2_O) m^−2^ s^−1^), water use at the vegetative phase (VWU; ml H_2_O plant^−1^ day^−1^), biomass (total aboveground biomass g plant^−1^), and HI (harvest index %) of selected ecotypes

Ecotype	WUE_*i*_			VWU	Biomass	HI
	90% rSWC	40% rSWC	20% rSWC			
Sq‐1	4.5	5.57	10.8	9.4	1.1	16.2
Se‐0	4.3	5.76	9.7	9.8	0.6	3.3
Lp2‐6	4.3	6.64	9.1	8.9	1.1	14
Lz‐0	4.5	6.05	9.7	11	0.2	13.6
C24	5.9	6.96	11.9	7.4	0.8	16.9
CIBC	4.4	6.27	9.9	6.5	0.7	20.9
Ws‐2	4.3	5.95	9.8	8.2	0.6	12.7

### 3.5 PCA highlights trade‐offs between vegetative and reproductive biomass, and drought sensitivity and flexibility of drought response

**Figure 8:**
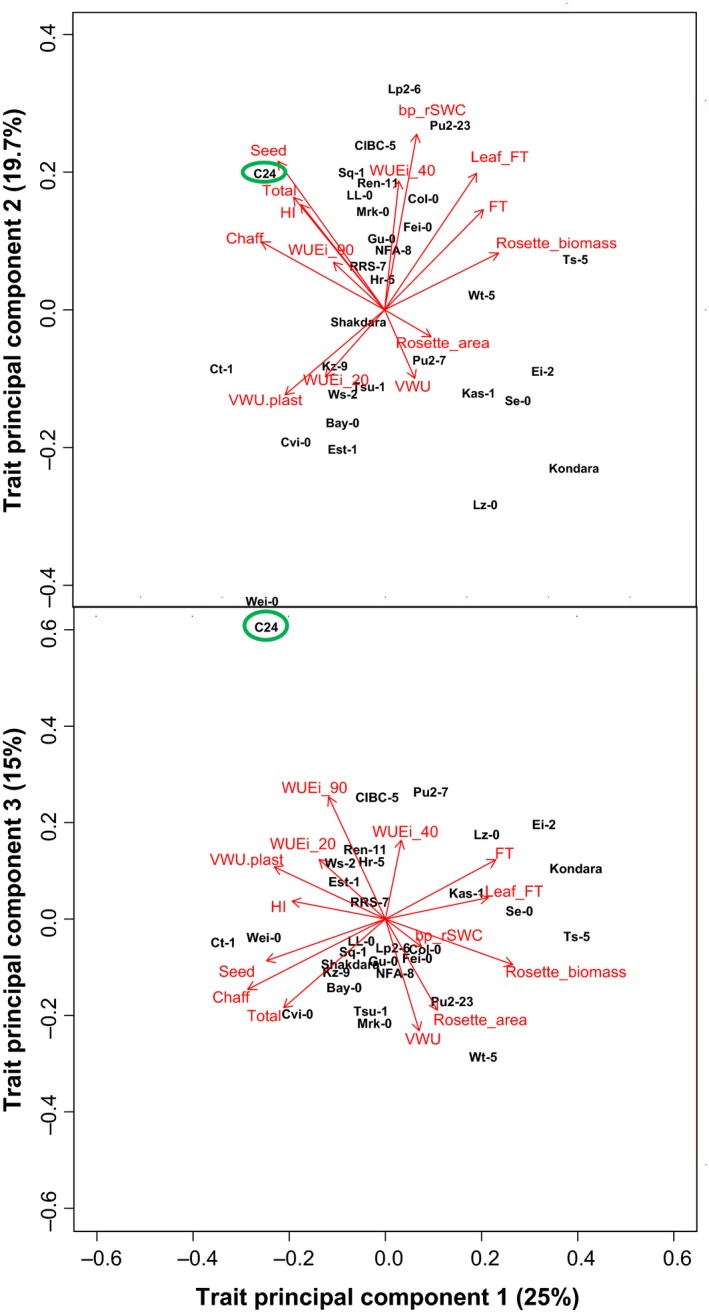
Principal component analysis of life‐history and water‐use‐related traits. Biplots displaying the loading onto trait PC1, PC2, and PC3 of 14 traits. The direction of the arrow represents the association of any particular trait parameter to trait PC1, PC2, and PC3, and the length of the arrow represents the strength of that relationship. The dispersion of individual ecotypes within the trait space is also displayed. FT—flowering time, Leaf_FT—leaf number at flowering, WUEi_90—WUE_*i*_ at 90% rSWC, WUEi_40—WUE_*i*_ at 40% rSWC, WUEi_20—WUE_*i*_ at 20% rSWC, bp_rSWC—breakpoint at rSWC determined by the segmented regression analysis, VWU—vegetative water use, and VWU.plast—VWU plasticity. The ecotype C24 is highlighted with a green circle

The above analysis highlighted the difficulties in identifying groups of ecotypes with similar water use and plant growth performance strategies. It was also evident that single trait correlation analysis could not account for the observed variation in many of the water use and biomass‐related traits. We therefore performed multivariate principal component analysis (PCA) on plant growth performance, WUE_*i*_ at 90%, 40%, and 20% rSWC, VWU, drought sensitivity (bp_rSWC), and VWU plasticity traits to detect potential underlying structures in the relationship between variables (Figure 8). PCA reduced the trait space to six statistically significant trait principal components (PCs), with the first three trait PCs explaining 60% of the overall variation (Table S7). The PCA demonstrated trade‐offs between reproductive biomass (seed and chaff) and vegetative biomass (rosette biomass) loading on trait PC1 (Figure 8, Table S7). The drought stress threshold (bp_rSWC) was in trade‐off with VWU plasticity loading onto trait PC2, while the different water‐use traits loaded most strongly onto trait PC3, with the per leaf area WUE_*i*_ (20, 40, and 90% rSWC) in trade‐off with the leaf surface area‐dependent transpiration (VWU). Ecotype dispersion within the trait space appeared to be more distinct along trait PC1 in the PC1/PC3 comparison and more distinct along trait PC2 in the PC1/PC2 comparison (Figure 8). This reflects the variation in biomass and plant development, as well as the differences in drought stress responses for the different ecotypes (Figure 8).

### 3.6 Potential for mapping water‐use traits

Genetic and phenotypic variances were calculated using GLMMs (see Section 2), to estimate broad‐sense heritability (*H* ^2^) (Table 1; Houle, 1992; Wagner & Altenberg, 1996). We observed significant *H* ^2^ for 32 of the 35 phenotypic traits measured as part of the SD experiments (Table 1). The *H* ^2^ of the primary fitness‐related traits, namely fitness (seed yield) and chaff, was comparatively lower than traits known to have a strong genetic basis, such as flowering time. Significant genetic variation existed for VWU, drought sensitivity (bp_rSWC), VWU plasticity and development and biomass parameters. We conclude that variation for these traits has a genetic basis (Table 1) and could therefore be discernible through the employment of appropriate genetic mapping approaches.

## 4 DISCUSSION

### 4.1 A short dehydration experiment provides insights into drought response strategies and water use

We have demonstrated highly significant phenotypic correlations between related physiological traits (Table S4). The increase in WUE_*i*_ under drought conditions was driven by a reduction in stomatal transpiration, which confirmed previous studies where stomatal limitations were the main driver of the reduction in carbon assimilation in Arabidopsis (Easlon et al., 2014; Kenney, Mckay, Richards, & Juenger, 2014; Masle, Gilmore, & Farquhar, 2005). However, there were no correlations between the physiological and plant growth‐related traits (Figure S4), which indicated that instantaneous gas exchange measurements failed to account for the impact of variation in environmental conditions, nor do they provide a proxy measure of integrated *A* over the entire lifetime of the plant (Driever, Lawson, Andralojc, Raines, & Parry, 2014; Lawson, Kramer, & Raines, 2012; Long, Zhu, Naidu, & Ort, 2006). However, the lack of correlation between rosette biomass and rosette area (Table S4), suggested that ecotypes with a tighter rosette and more leaf overlap may have diminished the effective photosynthetic surface, compared to the structural carbon investment into the leaves. This in turn may have an impact on the efficiency with which the plant uses the acquired carbon.

Similarly, root architecture plays an important role in adaptation to environmental conditions. Both root depth and density play a major role in optimizing water uptake depending on the hydrological conditions (Czyz & Dexter, 2012; Falik, Reides, Gersani, & Novoplansky, 2005). Due to the relatively small pot size (see Material and Methods) in our experiments, roots are likely to have been pot bound at the initiation of dehydration, potentially resulting in shorter and more branched roots, aiding water uptake under dry conditions (Poorter, Bühler, Van Dusschoten, Climent, & Postma, 2012). Natural variation of the root architecture under soil drying conditions has not been studied extensively in Arabidopsis; however, it has been shown that root impedance generally leads to reduced leaf expansion and may impact water status due to a reduced soil water holding capacity and faster drying rates (Bechtold et al., 2010). In our experiments, absolute VWU clearly depends on vegetative biomass (Figure 3d), and consequently, pot‐bound roots could have overall impacted on plant growth potentially underestimating VWU for some of the larger ecotypes. However, previous experiments in larger pots at approximately 1.5 to 1.3 g/L biomass to soil volume ratio found similar relationships with regard to water use and biomass allocation (Bechtold et al., 2010, 2013). Generally, relative performance for water use and biomass distribution was consistent between small and larger pot experiments.

We estimated absolute VWU based on a linear regression of the short dehydration response (Figure S1a). However, due to the “kink” in the dehydration profiles, which was different across ecotypes, we were also aiming to identify the exact breakpoint timing (rSWC at which plants greatly diminished their whole‐plant transpiration; Figure S2a,b) and the regression parameters of the different slopes. Segmented regression is advantageous for this type of analysis, as classical nonlinear methods, such as polynomial regression, regression splines or nonparametric smoothing, are not suitable either because the breakpoints are fixed a priori (regression splines), or are not taken into account (smoothing splines and polynomial regression; Muggeo, 2003).

Absolute VWU was positively correlated with rosette area (Figure 3d), and to account for this strong dependence, the segmented regression analysis allowed us to calculate drought response and drought sensitivity parameters that were effectively dimensionless and reduced the contribution of the transpiration surface (Figure 4a, Figure 5a).

In particular, the breakpoint in the dehydration cycle and WUE_*i*_ plasticity were useful to evaluate the drought response strategy of the different ecotypes (Figure 4, Figure 5). Drought‐sensitive ecotypes closed stomata early during the dehydration period and consequently showed stronger plasticity in leaf‐level WUE_*i*_ parameters (Figure 4). Selecting genotypes with low *g*_s_ and WUE_*i*_ plasticity during stage 1 (90%–40% rSWC) and higher WUE_*i*_ plasticity during stage 2 (40% to 20% rSWC) may be a favorable strategy for enhancing drought tolerance in areas with intermittent drought stress. This would suggest that ecotypes with “low” WUE_*i*_ values under well‐watered conditions and high WUE_*i*_ values under drought conditions are less drought‐sensitive and should therefore be able to outcompete other ecotypes. Multitrait analysis identified a group of ecotypes (group V, Table S6) with opposing WUE_*i*_ under well‐watered and drought conditions; however, this did not translate into plant growth performance (Table S6).

Nevertheless, the segmented regression analysis allowed us to characterize drought response strategies while at the same time measure absolute VWU. Crucially, the relationships between slope 1, slope 2, and rosette area are both significant and positive (Table S4), and we therefore propose that deriving absolute VWU as the slope of the linear regression is suitable as a drought response testing mechanism. The relationship was more significant with respect to slope 1, which suggests that VWU in the initial phase was affected by growth modality. However, slope 2 was significantly associated with *E* and WUE_*i*_, suggesting that VWU was also affected by stomatal dynamics (Table S4). We believe that the use of the “un‐normalized” absolute VWU has its merit, as differences in absolute water use reflect the actual water requirements of a plant and therefore will impact life‐time water use. Selecting ecotypes based on this initial analysis could subsequently be studied in more detail for different components of TE, especially life‐time water use, which would require the use of automated plant phenotyping platforms (Halperin et al., 2017; Tisne et al., 2013).

Interestingly, the ecotype C24 remained highly unique with trait combinations that were not observed in any of the remaining 34 ecotypes (Tables S5 and S6, Figure 8). Furthermore, C24 harbors resistances to a number of other abiotic and biotic stresses (Brosché et al., 2010; Lapin, Meyer, Takahashi, Bechtold, & Van den Ackerveken, 2012; Xu et al., 2015), and it is this unique combination of many abiotic and biotic stress tolerances, without apparent penalty in reproductive biomass that make this ecotype of special interest for further study. However, a much larger screen using automated phenotyping should be considered for this task.

### 4.2 Plant growth performance and water use under short‐day conditions are not linked to plant development

Previous studies have shown a positive correlation between leaf‐level WUE (δ^13^C) and flowering time, indicating that plants with longer lifespans exhibit high leaf‐level WUE (Kenney et al., 2014; McKay et al., 2003, 2008). Contrary to this, we did not observe a positive correlation between flowering time and WUE_*i*_ under well‐watered conditions (Table S4; Figure S3a,b). We chose to grow our plants in short‐day conditions (see Section 2) during the vegetative growth stage, to mimic conditions experienced by facultative winter annuals, spring annuals, and rapid cycling accessions that grow during the winter and spring. In contrast, previous studies of this nature often involved the growth of plants under long‐day photoperiods (Bac‐Molenaar et al., 2015; Clauw et al., 2015; Dittmar, Oakley, Ågren, & Schemske, 2014; Kenney et al., 2014), which reproduce the maximal day lengths experienced by Arabidopsis accessions that grow during the summer in Central Europe. Since Arabidopsis is photoperiod sensitive, extended day lengths substantially reduce the time to floral transition, reduce overall lifespan, and result in smaller rosettes and diminished leaf area (Martin, Tauer, & Lin, 1999; Menendez & Hall, 1995; Michaels & Amasino, 1999; Ngugi, Austin, Galwey, & Hall, 1996; Ngugi, Galwey, & Austin, 1994; Sayre, Acevedo, & Austin, 1995; White, Castillo, & Ehleringer, 1990). Under these long‐day conditions, any accession having a relatively shorter lifespan would indeed exhibit higher water‐use efficiency but this is clearly not a predictor of performance under short days.

Flowering time as a life‐history or fitness‐associated trait showed no positive correlation with final seed yield (Figure 8, Table S4). This suggests that flowering may be an important survival/fitness trait (Kenney et al., 2014; Schmalenbach, Zhang, Reymond, & Jiménez‐Gómez, 2014; Willis, Ruhfel, Primack, Miller‐Rushing, & Davis, 2008), but does not necessarily maximize productivity through remobilization of resources into inflorescences. The trade‐offs observed between flowering time, VWU and both vegetative and reproductive biomass (Figure 8), and the division of ecotypes along trait PC1 indicating that ecotypes with high vegetative biomass tend to have lower reproductive biomass substantiate this argument. Similar trade‐offs were observed in the perennial species *Arabidopsis lyrata* where populations increased their reproductive output while reducing vegetative growth, independent of flowering time (Remington, Leinonen, Leppälä, & Savolainen, 2013). Interestingly, in crops, this trade‐off has been actively selected for by reducing vegetative biomass to maximize yield (Sanchez‐Garcia, Royo, Aparicio, Martin‐Sanchez, & Álvaro, 2013).

### 4.3 Lessons from Arabidopsis research for improvement of water use in crops?

Efforts to dissect the genetic basis of fitness, water use, and drought resistance (Levitt, 1980) in crops are often based on proxy traits that are perceived to be associated with fitness, such as plant architecture, vegetative biomass, and flowering time (Younginger, Sirová, Cruzan, & Ballhorn, 2017). Many of these proxy traits are known to be less sensitive to environmental conditions have high heritabilities and are fast and easy to evaluate (Cai, Ye, Zhang, & Guo, 2014; Jiaqin et al., 2009). For example, the high throughput estimate of *A*/*g*_s_ as δ13C has been successfully used to screen for improved water‐use efficiency in many different plant species ranging from model to crop species (Campitelli, Des Marais, & Juenger, 2016; Christman, Donovan, & Richards, 2009; Jiaqin et al., 2009; Juenger et al., 2005; Korves et al., 2007; Rosas et al., 2014; Ruts, Matsubara, Wiese‐Klinkenberg, & Walter, 2012; Suter & Widmer, 2013; Todesco et al., 2010; Verslues & Juenger, 2011). The most consistent relationship between *δ* ^*13*^ *C* and wheat yield has been found in environments with high soil water status, where plants with high *δ* ^*13*^ *C* can grow faster and produce higher biomass under water‐replete conditions (Condon et al., 2002, 2004; Fischer et al., 1998). It has therefore been argued that many crop species selected for high yields have been bred without regard for the economy of water use and therefore often fail to optimize stomatal behavior under yield‐limiting growth conditions (Fischer et al., 1998). The selection for specific traits is in contrast to the concept of phenotypic integration, which describes patterns of inter‐trait correlations that define differences and trade‐offs, and provide an explanation on how phenotypes are sustained by the relationships between these traits (Nock, Vogt, & Beisner, 2016; Schlichting, 1989). It has been suggested that by selecting individual traits (i.e., yield), reductions in phenotypic integration have occurred in many crops, which may affect the possibilities of improving modern crops to deal with climate change (Milla, Morente‐Lopez, Alonso‐Rodrigo, Martin‐Robles, & Stuart Chapin, 2014).

We therefore reason that Arabidopsis as undomesticated species has maintained phenotypic integration and the trade‐offs between stomatal and whole‐plant water use as well as the different biomass parameters might reflect traits associated with adaptation to environmental conditions that have been lost in highly domesticated species. Arabidopsis‐focused studies therefore yield information regarding the importance of key traits and their relationships essential for water use and productivity in a species that has not been selected to disregard water availability in pursuit of maximal rates of photosynthesis and productivity. Consequently, genes identified through phenotyping and mapping of the existing natural variation of VWU in Arabidopsis may represent useful candidates for the improvement of stress tolerance and water use at least in closely related *Brassica* crops (Bechtold et al., 2013).

## 5 CONCLUSION

Our study has demonstrated that a short dehydration cycle followed by a segmented regression analysis has potential as a screening tool for plant water use and drought response strategies. We believe that it could be very useful in larger and rapid screens for assessing drought response parameters, when automated phenotyping facilities are not readily accessible. Using this approach, we were, however, unable to identify ecotypes that mirrored the behavior of C24 under well‐watered and drought stress conditions. Therefore, in order to identify the underlying genetic basis for these trait combinations, either a much larger ecotype screen or the use of mapping populations need to be considered.

## AUTHOR CONTRIBUTIONS

J.N.F., O.B., and U.B. performed and analyzed all experiments. J.N.F., T.L., M.H., and U.B. planned and designed the experiments. U.B., O.B., and J.N.F. wrote the manuscript with input from all the authors.
