# Supplementary material for: Natural variation of life‐history traits, water use, and drought responses in Arabidopsis
Source: Plant Direct. 2018 Feb 1;2(1):e00035. doi: 10.1002/pld3.35 (PMC6508493; doi:10.1002/pld3.35)
Supplement: Supplementary file 1 [file PLD3-2-e00035-s001.pdf]

a

b

c

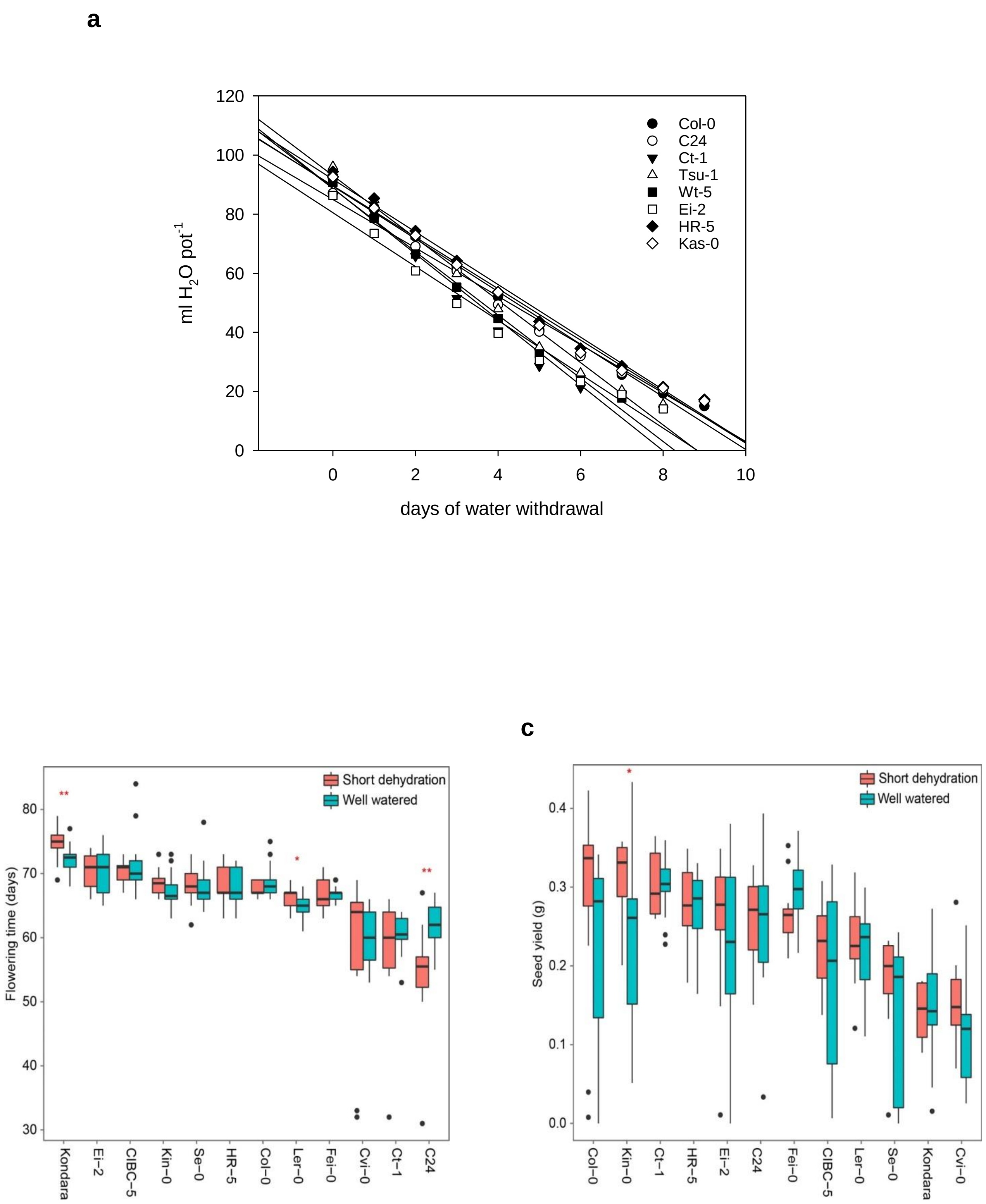

Figure S1, Ferguson et al 2017

Figure S1: a Example of a progressive soil drying experiment of 8 ecotypes. Vegetative water use (VWU) was calculated as the overall slope of the linear regression, n = 15. **b** Boxplots describing the variation for flowering time for a short dehydration (red) and well-watered experiment (blue). **c** Boxplots describing the variation for a short dehydration (red) and well-watered experiment (blue). Red asterisks denote significant difference between the same ecotype grown under short dehydration and well-watered conditions, where *** = $P < 0.001$, ** = $P < 0.01$, and * = $P < 0.05$; n = 15. The bold line in the centre of the boxplots represents the median, the box edges represent the 25th (lower) and 75th (upper) percentiles, the whiskers extend to the most extreme data points that are no more than 1.5x the length of the upper or lower segment. Outliers are data points that lie outside the 1.5x interquartile range both above the upper quartile and below the lower quartile.

a

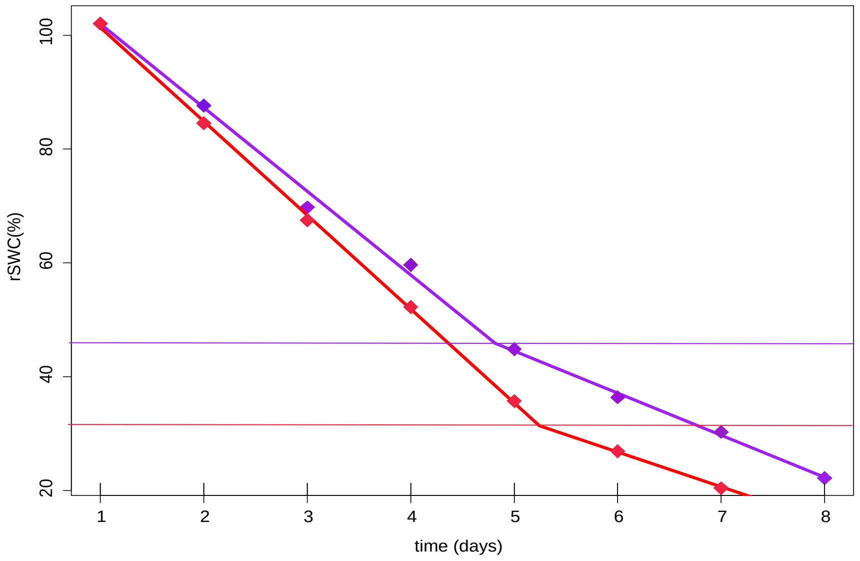

b

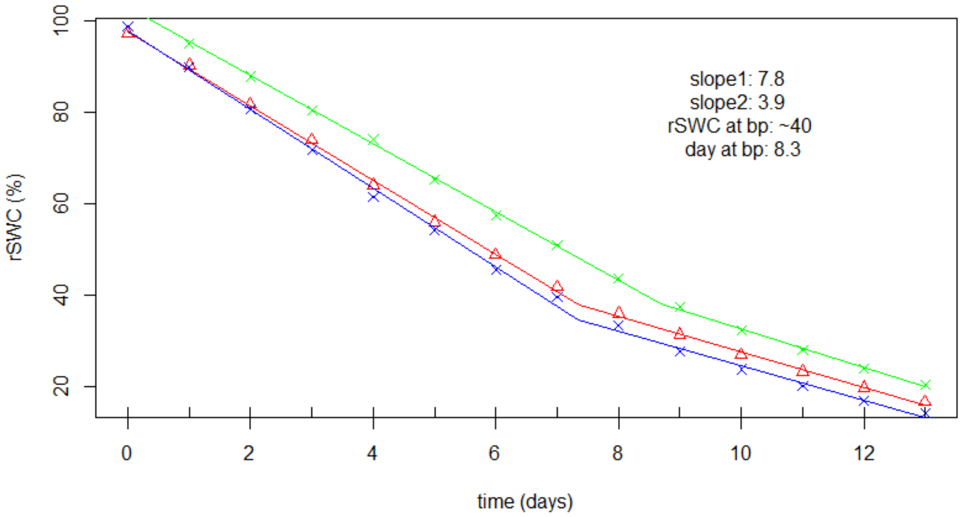

Figure S2. a Example of a segmented regression analysis for two contrasting Arabidopsis ecotypes. Segmented regression analysis was carried out on 15 replicates for each ecotype**. b** Calculation of the breakpoint for a dehydration experiment carried out in Col-0 published in (Bechtold et al 2016). The average slope parameters and breakpoints are provided on the graph, n = 30. The average breakpoint at 40% rSWC (~day 8) coincided with the substantial transcriptional changes (Bechtold et al 2016). In order to calculate VWU, rSWC (%) was converted into ml water per pot, based on the average volume of water required to saturate pots.

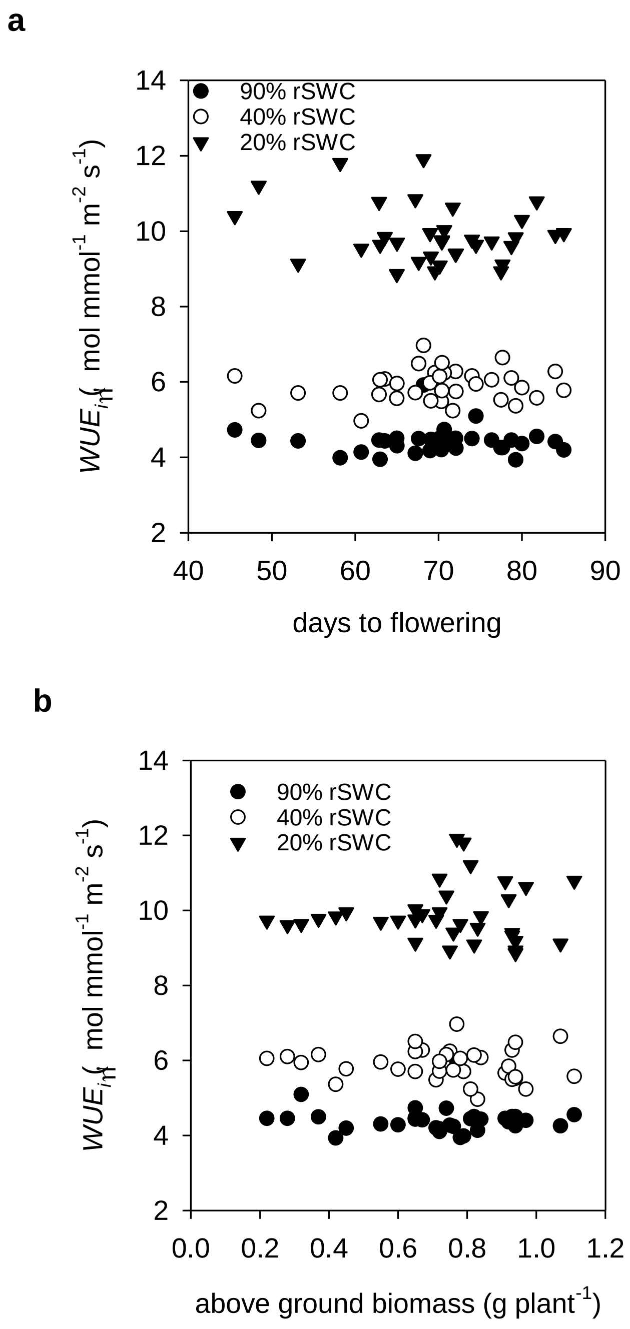

Figure S3. a Association between days to flowering and instantaneous water-use efficiency (*WUE_i*) at 90% rSWC. **b** Association between above ground biomass and *WUE_i* at 90% rSWC. No significant associations were observed.

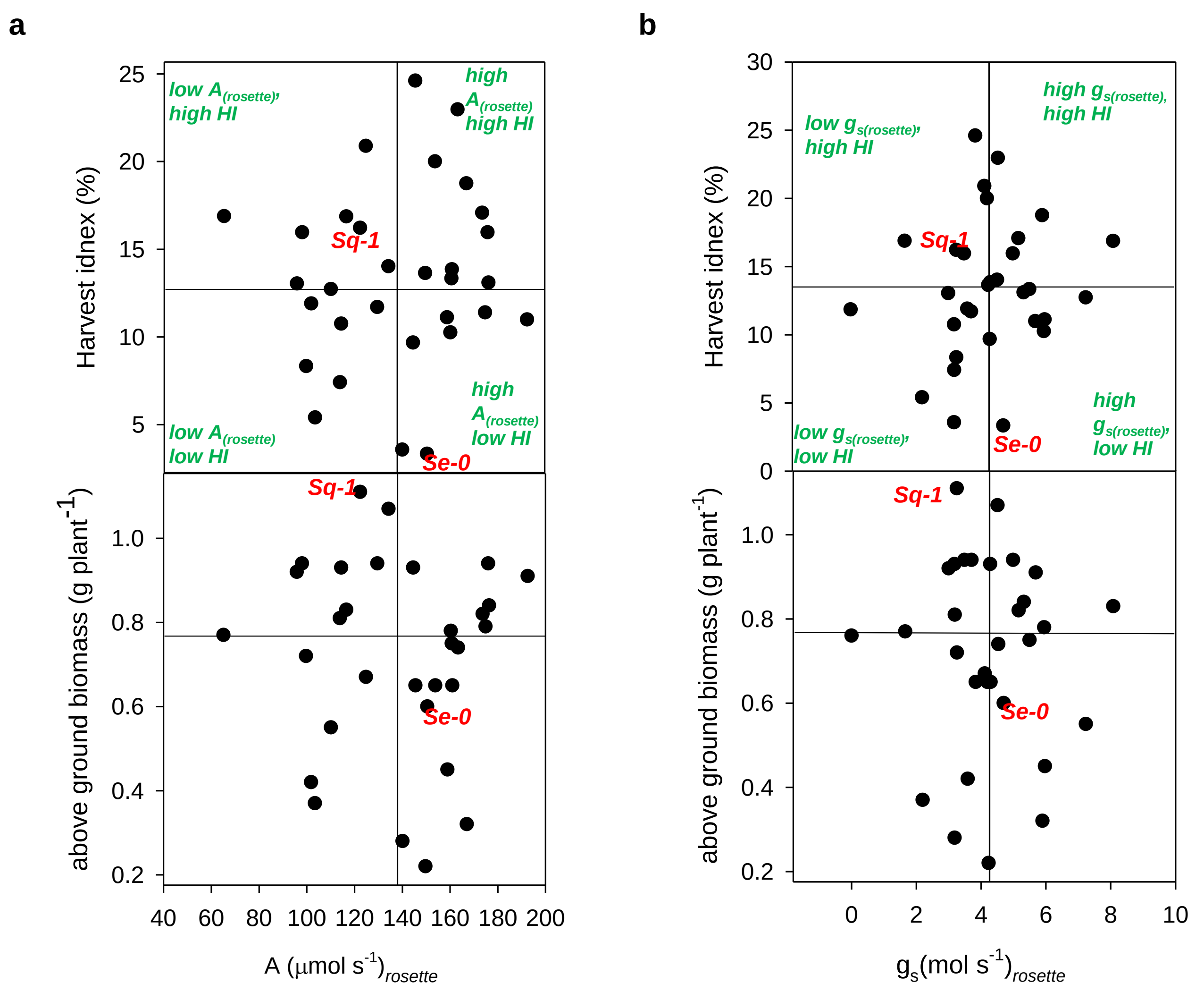

Figure S4: Impact of *A* and g_s on biomass production and allocation **a** Identification of accessions with greater values of rosette $A_{(rosette)}$, above ground biomass and harvest index. **b** Identification of accessions with greater values of $g_{s(rosette)}$, above ground biomass and seed harvest index. Accessions with high harvest index, biomass and low $g_{s,(rosette)}$ (presumed low water use) are in the upper left hand quadrant. The lines dividing the space into quadrants represent the median for the each trait.
